# Self-Assembly of Atomically Precise Nanoclusters: From Irregular Assembly to Crystalline Assembly

**DOI:** 10.3390/nano13182551

**Published:** 2023-09-13

**Authors:** Rodolphe Antoine

**Affiliations:** Institut Lumière Matière UMR 5306, Université Claude Bernard Lyon 1, CNRS, Université Lyon, F-69100 Villeurbanne, France; rodolphe.antoine@univ-lyon1.fr; Tel.: +33-(0)-787-098-059

The persistent efforts toward achieving superior properties for assembled nanoscale particles have been held back due to the resulting polydispersity associated with colloidal routes of synthesis [1]. The emergence of ligand-protected atomic clusters seems to be a solution to this limitation [2]. In this case, the ligands stabilizing the clusters are highly reactive in nature and thus provide a facile avenue for the “ligand-mediated spatial organization of nanoclusters” [3]. Further, the most important characteristic of nanoclusters, which distinguishes them from other classes of nanomaterials, is that atomic clusters are imbued with structural integrity. Even polydispersed nanoclusters may be purified following regular purification techniques, and the precise chemical formula of the nanoclusters may be deciphered thanks to mass spectrometry [4]. Thus, unlike other forms of nanoscale particles, a dispersion of atomic clusters typically constitutes of structurally and chemically related species. Controlling the size of nano-constructs is of the utmost importance to obtain a nano-theranostic device, as outlined by Mellor et al. [5]. Hence, the study of chemical reactions toward achieving complex nanostructures in a controllable manner could yield self-assembled nanoclusters with multiple functions and collective properties [6], widening their application potential [7].

The aim of this Special Issue on “Self-Assembly of Atomically Precise Nanoclusters” was to provide a unique international forum aimed at covering a broad description of the various approaches developed for assembling nanostructures (in particular nanoclusters) into higher ordered structures in various dimensions.

Synthetic routes are at the heart of supramolecular chemistry. Innovative strategies, inspired by colloidal routes, lead to 3D assemblies of nanoscale materials. Kim et al. [8] used the evaporation of a fine fountain pen to form 3D colloidal assemblies composed of nanoparticles. This route could be widely applied as a simple fabrication tool in order to explore complex metamaterials constructed of nanoparticles, as this method is highly flexible in varying the shape as well as the composition ratio of self-assembled structures. Also, noble metal nanoparticles provide a reaction platform that plays dual roles in the formation of ligand-protected gold nanoclusters. Cheng et al. used the surface of nanoparticles, with four different shapes, to reduce gold ions and to attract capping ligands [9]. Alternative synthetic routes can use a pulsed plasma approach for the production and surface deposition of silicon nanoclusters (SiNCs) for a size of about one to two nanometers. The as-produced one-to-two-nanometer SiNCs can assemble to form much larger “superclusters” with a size of tens of nanometers. These superclusters possess extremely high permanent electric dipole moments that can be exploited to orient and guide these clusters with external electric fields, opening the path to the controlled architecture of silicon nanostructures [10]. Amorphous or irregular assemblies are also of great interest since they are found in natural processes. The growth of so-called amorphous calcium carbonate nanoparticles into micro-to-millimeter scale crystals is the key step in the formation of the shells and exoskeletons of most marine animals. Understanding the mechanism by which these nanoparticles self-assemble is essential to better understand the effects of climate change on marine life. In this spirit, Clark et al. explored the kinetics of aragonite formation from solution via amorphous calcium carbonate [11].

Ligands, which protect the nanoclusters, play a vital role for stability and for enhancing their properties. In particular, ligands may affect the intramolecular configuration, intermolecular packing, and optical properties of metal nanoclusters. Wu et al. [12] used a Ag_22_ nanocluster template to address the effects of surface modification on intracluster constructions and intercluster packing modes, as well as the properties of nanoclusters or cluster-based crystallographic assemblies. At the supramolecular level, the regulation of intramolecular and intermolecular interactions in nanocluster crystallographic assemblies rendered them CIEE (crystallization-induced emission enhancement) active or inactive nanomaterials. Silver nanoclusters (Ag NCs), as a material with good aggregation-induced emission and biocompatibility, have been widely used in the field of luminescence. However, there are few studies on inducing Ag NCs to obtain chirality through supramolecular self-assembly. Therefore, realizing the chiral self-assembly of Ag NCs is still an urgent problem to be solved. Wang et al. [13] used silver NCs (Ag_9_-NCs, [Ag_9_(mba)_9_], where H2mba = 2-mercaptobenzoic acid) and peptide DD-5 (polymerization of five aspartic acids) to obtain highly ordered fluorescent nanotubes through supramolecular self-assembly. With the introduction of DD-5, the Ag9-NCs AIE effect was triggered and the chirality of the peptide was successfully transferred to the supramolecular assembly, resulting in an assembly that possessed chirality and good circularly polarized luminescence characteristics. Proteins, and in particular bovine serum, are good templates to stabilize and provide highly fluorescent bioconjugates [14]. It is well-known that the deuterium–hydrogen isotope effect causes significant changes in the folding–unfolding processes of proteins. Fehér et al. [15] showed that heavy water, compared to normal water, induces stronger effects in both global and fine structures of bioconjugates and that these changes bring a significantly increased red fluorescence than that observed in normal water.

To conclude this overview on the papers published in the Special Issue “Self-Assembly of Atomically Precise Nanoclusters”, I am confident that the readers will enjoy these contributions and may be able to find inspiration for their own research within this Special Issue. This series of manuscripts on this topic will give maximum impact and allow workers in other research areas to apply the same methodologies in understanding the mechanisms of self-assembly in their systems.
